# Remarks on the Cause of Purpura, and Its Distinction from Scurvy

**Published:** 1809-04

**Authors:** Robert Bree


### *Remarks on the Cause of Purpura, and its Distinction from Scurvy.* By Robert Bree, M. D. F. R. S.

SOME ingenious observations on Purpura, by Dr. Parry, appeared in the last Edinburgh Medical Journal, which had been preceded by remarks in the London Medical Journal, where the Editors considered purpura and scurvy to have a near affinity. The object of these observations was to show a distinction between these diseases, contrary to general opinion, and to that stated in the elaborate work of Dr. Willan, who thinks that purpura pertains to scurvy.

I think it probable, that the following cases may afford useful inferences, and, perhaps, elucidate the nature of their distinction.

Many years ago, I was consulted by a woman of 60 years of age, who was covered with numerous patches of purple over the breast, arms, and legs. The intermediate spaces were specked with petechiæ. This appearance of the skin had begun to shew itself three weeks before, and gradually increased till I saw her. In the same proportion a debility of the whole system had increased, which now bore the character of a paralytic weakness, the right side being much more infirm than the left. She had had vertigo for seven weeks, and the confusion of her head was violently augmented by motion. She had been affected with symptoms of apoplexy a year before this illness, and had been relieved by the usual means. On the present occasion, the appearance of petechiæ and purple vibices, had deterred a respectable practitioner from resorting to bleeding. Her pulse was 70, and full. It was not, however, hard. She was of great bulk; the eyes were suffused with red, and her speech was much impeded. She was bled, and afterwards purged with jalap and calomel. The blood was dense, but not inflamed. She had feelings of internal relief, and the pulse rose to 76 after her bleeding.

Two

Two days afterwards, as the head was still oppressed, and the power of voluntary action not improved, although the purple vibices remained, I directed a repetition of the bleeding, and in four days more she was cupped. She acquired strength under these evacuations of blood and considerable purging, and in a week the purple spots were gone. She probably lost twenty-five ounces of blood. The symptoms of paralysis left her gradually, as the skin recovered its healthy colour.

A year and a half afterwards, on the approach of winter, the same concurrence of symptoms, in less degree of force, again attacked her, but was perfectly removed by the same means.

### CASE II.

An elderly lady had been several times affected with slight symptoms of paralysis, which had given way to purges of calomel; she had been oppressed with bile, and relieved by copious discharges from the bowels in the early period of her complaint.

The organs of speech were then occasionally affected, with some confusion of the head, and a full and slow pulse. She was at last seized with a diarrhœa, and considerable general weakness. The pulse was still full, and the head confused; the diarrhœa was at first considered critical, and treated by calomel and rhubarb; but this plan was soon changed, from a consideration of the state of the pulse, and the pressure on the brain. She was bled to ten ounces; the diarrhœa was immediately stopped, and by attention to the state of the head and bowels, she recovered her former health.

### CASE III.

A Gentleman of Kensington, of middle age, plethoric, and sedentary, but active in his mind, was seized in the last month with a slight apoplexy, that suspended his senses for a short time in the night. I saw him the next morning. His breast was then thickly beset with petechiæ. His eyes were suffused with red; his pulse was full, and firm, but not hard nor throbbing. He had not complained of his head, but of the torpor and involuntary motions of his lower limbs; his left leg being sleepy, and his right being drawn by cramps of the muscles, which was now his most distressing complaint. He had not been conscious of the extent of the disorder, but the spasms in his legs had awakened him before the loss of his senses took place.

He was bled to ten ounces, and purged. The

The Medical gentleman, who had attended to his case with great ability, observing the petechiæ, hesitated on bleeding. His doubt might be naturally strengthened by the character of the patient's habit, which, though full, was relaxed and nervous; but the evacuation soon relieved the general state, and the petechiæ disappeared.

Three weeks afterwards, this patient having exposed himself to cold winds, was attacked in the middle of the night by the same train of symptoms in greater violence. He came out of one seizure, but directly experienced a second; in the absence of his senses he was bled largely, and came to himself. His legs were affected with torpor, cramps, and involuntary action, as in the first attack. The petechiæ again appeared very numerously upon his body, and continued two days, when they left him, under the treatment of large bleeding, a saline camphorated draught every six hours, and a purge with jalap and infusion of senna every morning.

My inferences from these facts are not exactly such as coincide with the pathology of Dr. Parry, but they do not oppose the reasoning of that able physician; and I think it probable, they may add to his general evidence of the distinction between purpura and scorbutus.

The apoplectic affection that appeared in combination with purpura, in the 1st and 3d cases, leads me to conclude, that purpura may arise from compression of the brain, giving occasion to the want of a contractile power in the fibres of the extreme vessels.

The second Case, I consider explanatory of this pathology. It is not a solitary instance of the relaxation of the excretory vessels of the liver and alimentary canal from compression of the brain. Every physician of experience must have observed, that the alimentary canal is occasionally slowly affected by purgative medicines from want of sensibility, and that at other times it is in a state of looseness from the same cause; and, that lessening the pressure of blood upon the brain, is the best means of restraining diarrhœa, in some instances, and of removing costiveness by restoring the irritability of the coats of the canal in others.

I conceive, that the excretory and exhalent vessels were for a time without the power of due contraction in this case, but that the energy of the nervous system was restored by bleeding, and renewed the sensibility of their fibres.

The 1st and 3d Case, furnish no data for reasoning differently respecting their pathology. There is no certain conclusion to be drawn of an over distension of any of the these extreme vessels, unless this be a necessary effect of their paralytic condition, and the want of contractile power in their fibres.

As to the treatment of cases of this kind, I consider depletion to be necessary, from the local fullness of the brain, and not from the existence of active hæmorrhage.

If the vessels of the head be sufficiently emptied, and kept free from distension, the extreme arteries may recover their contractile power, although the system at large should preserve its original state. The gaping of these extreme vessels must be the effect of their passive state, and pressure at the origin of the nerves should be taken off, before other modes of relief, by stimulants or tonics, if required, are applied.

I do not presume to deny the probable existence of an active state of the general vascular system in other cases of purpura; but, according to my observation, this active state is not necessary to the production of purpura, any more than it is to that of scurvy.

Yet, the distinction between purpura and scurvy should be clearly established for the purposes of practice, and I conceive it may be sufficiently shewn in the predisposition of the body. The sponginess of the gums, and the bleeding from them, do not attend purpura, and these symptoms indicate a more general and chronic debility of the whole system. There is no urgent local affection in scurvy to demand a primary treatment.

If, in Scurvy, the debility of the whole system, the length of time that it has had for its growth, and the nature of the depressing causes that brought it on, could really occasion distension of the vessels of the head, this state would not be an object of exclusive treatment. The two diseases must have attained different degrees of debility, even in this view of their affinity; and in such different stages, a distinct treatment is requisite for each.

The mineral acids, Peruvian bark, and oxygen, would not be well timed in purpura, before the brain was relieved from pressure. In scurvy, they could not be too soon adopted, since the powers of the whole system, and the crasis of the blood and its exciting property are weakened or destroyed.

I think from these premises, that scurvy may include purpura, without pressure on the brain as a remote cause; but that purpura is not necessarily scurvy, any more than a symptom is a disease.

Purpura, as well as scurvy, is a disease of debility, but but it may be treated successfully by evacuants; and it therefore furnishes new testimony, if any were wanted, to prove how useless names of diseases are to the experienced physician, when he comes to the consideration of a chronic complaint.

*London, March* 17, 1809.

